# Accurate and Cost-Effective Micro Sun Sensor based on CMOS Black Sun Effect †

**DOI:** 10.3390/s19030739

**Published:** 2019-02-12

**Authors:** Rashid Saleem, Sukhan Lee

**Affiliations:** Intelligent System Research Institute, Sungkyunkwan University, Seobu-ro 2066, Jangan-gu, Suwon-si, Gyeonggi-do 16419, Korea; mrashid@skku.edu

**Keywords:** sun sensor, black sun effect, icosahedron configuration, sensor fusion

## Abstract

An accurate and cost-effective micro sun sensor based on the extraction of the sun vector using a phenomenon called the “black sun” is presented. Unlike conventional image-based sun sensors where there is difficulty in accurately detecting the sun center, the black sun effect allows the sun center to be accurately extracted even with the sun image appearing irregular and noisy due to glare. This allows the proposed micro sun sensor to achieve high accuracy even when a 1 mm × 1 mm CMOS image sensor with a resolution of 250 × 250 pixels is used. The proposed micro sun sensor is implemented in two application modes: (1) a stationary mode targeted at tracking the sun for heliostats or solar panels with a fixed pose of single image sensor of 1 mm × 1 mm × 1.74 mm in size and (2) a non-stationary mode targeted at determining the orientation of moving platforms with six sensors on the platform, which is configured in an icosahedron geometry of 23 mm × 23 mm × 12 mm in size. For the stationary mode, we obtained an accuracy of 0.013° by applying Kalman filter to the sun sensor measurement for a particular sensor orientation. For the non-stationary mode, we obtained an improved accuracy of 0.05° by fusing the measurements from three sun sensors available at any instant of time. Furthermore, experiments indicate that the black sun effect makes the precision of sun vector extraction independent of the sun location captured on the image plane.

## 1. Introduction

**Figure 1:**
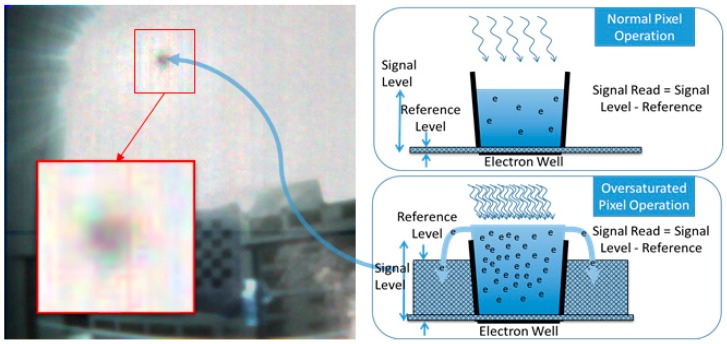
**Left**: Sun captured by a NanEye camera; **Right**: Oversaturation in a CMOS Image Sensor causes electron overspill that increases the reference voltage, resulting in the output signal being “near zero”.

One of the challenges in astrophysics, while capturing the images of a celestial star, is the optical phenomenon called limb darkening [1]. It is an optical effect seen in celestial objects, such as the sun, where the center appears to be brighter than the edge or limb of the star. Thus, the sun center will be the brightest spot observable, often causing the incident image pixel to be oversaturated. When a CMOS image pixel is oversaturated, the phenomenon of electron overspill occurs, such that the output signal of the pixel is read as “near zero” if no proper compensation is provided, as shown in Figure 1. This is referred to as the “black sun” effect. Although black sun is an undesirable phenomenon that is removed in ordinary photometry [2], we use it to accurately and robustly determine the sun centroid in an image for the sun vector extraction in image-based sun sensors. Sun sensors are widely used in space applications [3,4] but are not limited to them. Applications, such as alternative power generation relying on solar panels [5] and heliostats [6] on earth, also require accurate solar tracking to provide maximum throughput.

Sun sensors are based on microelectromechanical systems (MEMS) and camera image sensors. MEMS-based sun sensors [7,8] have a mask with small pinhole apertures placed in front of the active pixel sensor (APS). MEMS-based sun sensors offer high accuracy with the tradeoff of a small field-of-view (FOV). To increase the FOV, MEMS-based sun sensors use multiple masks or sensors, which increases the overall cost. For instance, a commercially available MEMS-based sun sensor [9] having an accuracy of 0.3° can cost over €10K. XDSS [10] fabricated an accurate MEMS-based sun sensor with a large FOV by using a mask with a 13 × 13 aperture array, where each aperture provides 13° × 13° sub-FOVs, resulting in a 105° × 105° FOV with 0.05° accuracy. SENSOSAL, developed for CubeSat [11], employed five MEMS-based sensors—one with 0.05° precision and ± 6° FOV and four with 0.5° precision and ± 60° FOV. On the other hand, camera-based sun sensors focus on large FOVs, they have reduced cost and offer accuracies acceptable for the given task. Jet Propulsion Lab (JPL) [12] designed a sun sensor for the Mars Exploration Rover (MER) by using a charge-coupled device (CCD) monochrome wide-angle camera with a FOV of 120° × 84°. The sensor used a neutral density optical filter to reduce incoming light in such a way that it could capture the sun and compute the centroid with a precision of 1°. Deans et al. [13] used a 180° FOV fish-eye lens with off-the-shelf components to achieve a precision within 1° for 89.77% of the time. Barnes et al. [14] introduced a novel sun sensor using 17 light intensity sensors positioned uniformly over a hemispherical shape and were able to obtain solar azimuth and zenith precisions within 5° and 1°, respectively. While they achieved 360° coverage, their overall accuracy was considerably low and suggested adding more sensors to improve the accuracy. Liu et al. [15] extended this approach by using an array of nine CCDs with a 640 × 480 pixel resolution instead of light intensity sensors to obtain a precision of 0.2° and 0.1° in azimuth and zenith, respectively. However, they arranged their cameras in such a manner that the sun captured should lay in the central image area to obtain higher accuracy. They also used the blooming of the CCD to their advantage by using blooming lines to supplement the sun vector extraction. Nevertheless, they observed that the error increases when sun image is captured at image boundaries due to increased distortion effect or when two blooming lines are close together; their proposed sensor is larger than 200 mm × 200 mm. Conventional instruments such as a MICROTOPS II sun photometer [16] can also track the sun by keeping the bright point of light from the sun in their crosshair, but with the tracking accuracy of up to 1°. The focus of this research is on filling the gap between MEMS and camera-based sun sensors by supplementing the simplicity, cost-effectiveness, and wide FOV of camera-based sun sensors with the accuracy and small size of MEMS-based sun sensors. We achieve this by proposing an approach for extracting the sun vector that will result in a wide-FOV, cost-efficient, and accurate sun sensor that uses off-the-shelf components for easy deployment.

In this paper, we present a cost-effective micro sun sensor by extracting the sun vector from image sensors accurately and robustly using the black sun effect, despite irregular and noisy sun image due to glare. A short version of this paper with preliminary results was presented at IEEE Sensors 2017 [17]. Extensive testing is presented in this paper along with new observations. We found false positives being reported by the centroid algorithm which was addressed by adding additional checks to it. Additional performance comparison of the centroid algorithm with the conventional centroid method was also conducted. Unlike other conventional image-based sun sensors, we observed that our black sun effect based sun vector measurement error or variance is independent of the location of the sun captured on the image plane. To evaluate the sensor’s performance purely, we ignored the transformation between topocentric and sensor frame and evaluated the sun vector measurement in the sensor frame. We demonstrate its performance based on two types of applications: a stationary application for heliostats or solar panels with a single image sensor configuration at the fixed pose and a non-stationary application for determining the orientation of a moving platform, such as the space rover with a multiple-image-sensor icosahedron configuration. The experiments indicate an improved accuracy of 0.013° is achievable in azimuth and elevation for the stationary application and 0.05° in azimuth and elevation for the non-stationary applications. The stationary application offers a FOV of 90° whereas the non-stationary application offers 360° with a fraction of the cost of MEMS-based sun sensors. 

## 2. Sun Vector Extraction

### 2.1. Camera Selection

**Figure 2:**
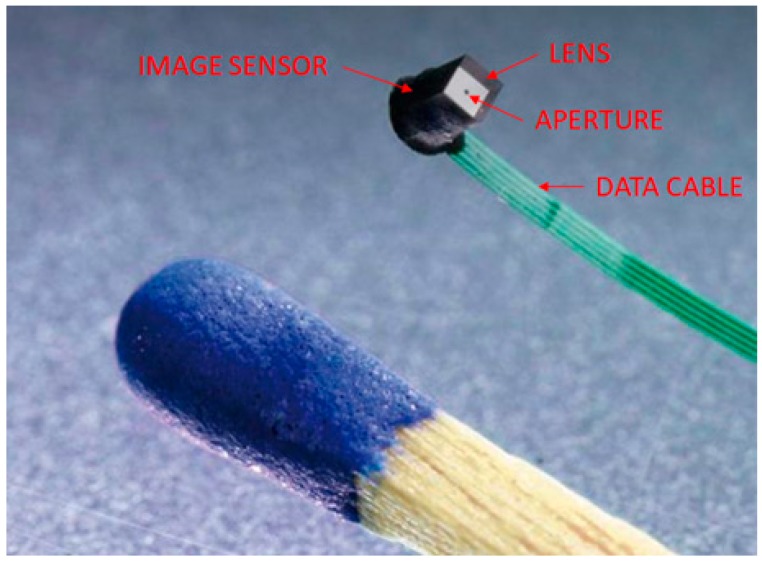
Awaiba NanEye 2D camera module compared with a matchstick for size (Source: CMOSIS, 2015).

For our study, we selected the Awaiba NanEye 2D camera [18], which is presently amongst the smallest CMOS cameras commercially available (Figure 2). The NanEye 2D was specifically designed to be used for an endoscopic application having a size of 1 mm × 1 mm × 1.74 mm with a resolution of 250 × 250 pixels and a 90° FOV. The camera module is supported by a Xilinx Spartan-6 FPGA frame grabber that is capable of capturing four cameras simultaneously at 42 to 55 fps. Not only does the size give us flexibility, but its ability to exhibit the black sun effect also helps us to achieve off-the-shelf component assembly for cost-effectiveness.

**Figure 3:**
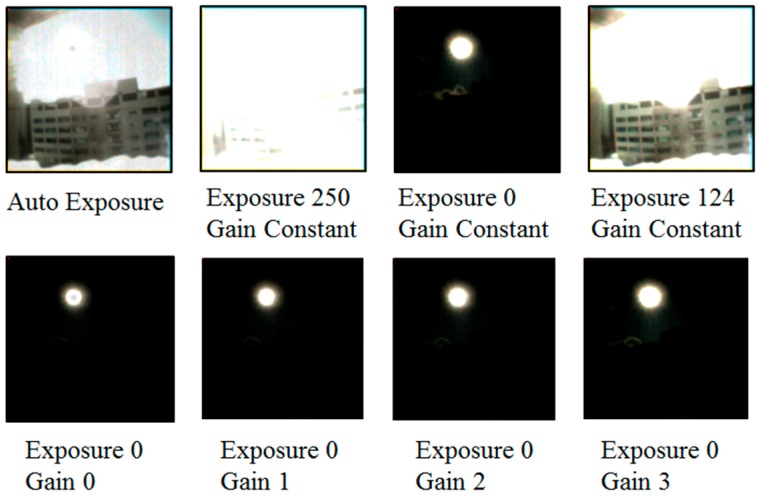
Visibility of the black sun with different camera parameters.

The black sun effect is an unwanted effect that most manufacturers remove through a post-image process by replacing the affected pixel value with the surrounding pixel value. Thus, it was very crucial to study what parameters could affect the appearance of the black sun in the NanEye camera. The chip-on-the-tip camera we used has an automatic exposure control integrated into it and allows the control up to 250 exposure steps. Each step exposure step decreases the exposure time by 90.8 ns (equivalent of the exposure time of one line). The image signal gain can also be set at four levels; i.e., gain 0 = −1.6 dB, gain 1 = 1 dB, gain 2 = 2.4 dB, and gain 3 = 6.5 dB. Figure 3 below shows the samples of different parameters.

### 2.2. Centroid Detection

**Figure 4:**
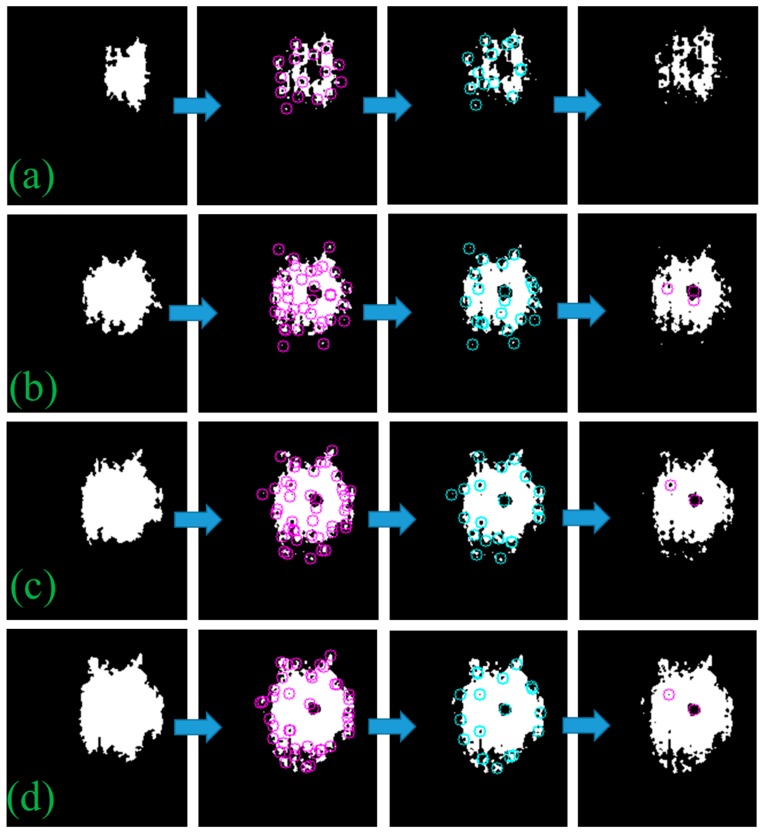
From left to right, a gradual step of decrementing the intensity by using the centroid detection algorithm to refine the corner points in each iteration is seen. Each row represents an iteration, if the loop size is 4 then (**a**) the pixel intensity is greater than the threshold +4; (**b**) the pixel intensity is greater than the threshold + 3; (**c**) the pixel intensity is greater than the threshold + 2; (**d**) the pixel intensity is greater than the threshold + 1.

**Figure 5:**
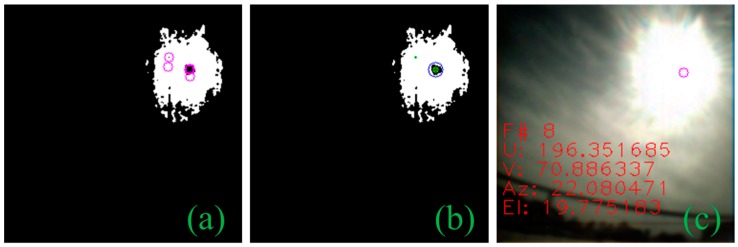
The final stage of the centroid detection algorithm after all iterations: (**a**) all potential black sun candidates; (**b**) points surviving between iterations; (**c**) black sun centroid after determining the point with the largest radius.

Based on the black sun effect in a CMOS imaging sensor, we were able to detect the centroid of the sun with its shape invariance. When the sun image is captured, the sun image segment has one area—the black sun—which has a different pixel intensity than that of the surrounding segments. In Figure 4, each of the rows shows the binary mask of the sun image captured by the sensor at different pixel intensity thresholds. The sun image segment is irregular and noisy, particularly in the case of Figure 4a, although the black sun spot appears inside the pixel mass but not necessarily at the center which causes conventional methods, such as Circle Hough transform (CHT) [19], to fail. To have a robust detection, in any case, we gradually decrease the intensity value from maximum pixel intensity to a certain level iteratively, thus, making it possible for the centroid positioning in tiny, noisy, and insufficiently conditioned segments. We first detect the strong corners in the binary mask and then mark their survival over multiple iterations. In each iteration, we create a binary mask with an intensity lower than that in the last iteration. The sub-accuracy of strong corner points is refined by using subpixels (i.e., hyperacuity). Corner points that appeared at the edge of the binary mask are removed based on distance from the boundary of the largest contour shape in the binary mask of that iteration. An additional check is made that the largest contour shape has an eccentricity of less than 0.9. Otherwise, some image artifacts were causing false positives due to the wrong selection of contour shape. In Figure 4, each row represents an individual iteration, and each column from left to right represents an incremental step within an iteration. After running all iterations, we have all the possible candidate points (Figure 5a), now we determine which points survived between iterations (Figure 5b) and then select the point that has the largest radius because the black sun will be the largest segment inside, as seen in Figure 5c. In addition, we had to perform an additional check that the black sun is at least a specific size. Because the algorithm was producing outliers more weight is given to the surviving points between iteration rather than the largest radius. The pseudocode for the proposed centroid detection algorithm is given in Algorithm 1.

**Algorithm 1.** Sun centroid detection using black sun detection**Input:** Captured Image1: Apply Gaussian Blur and convert to grayscale 2: Set *number of loop f* and *threshold = maximum pixel intensity -*
α (α: user-defined unsigned integer based on empirical sensor performance) 3: **while**
*f >= 1*
   3-1: Generate binary mask for pixels with *intensity > threshold + f*
   3-2: Find contour in the binary mask    3-3: Find the index of largest contour    3-4: Get strong corner points     3-5: Find subpixel    3-6: Save corner points inside the largest contour (with eccentricity < 0.9) away from edges    3-7: Accumulate surviving points between iterations     3-8: Decrement *f*
  **end while**
4: Accumulate corner points 5: **if** no surviving points then    Get accumulated corner point    Check for point with the largest radius > minimum radius   **else**
    Get surviving points    Check for point with the largest radius > minimum radius  **end if**
**Output:** Black Sun Centroid Coordinates $\left(C_{x},C_{y}\right)$


### 2.3. Performance Comparison

**Figure 6:**
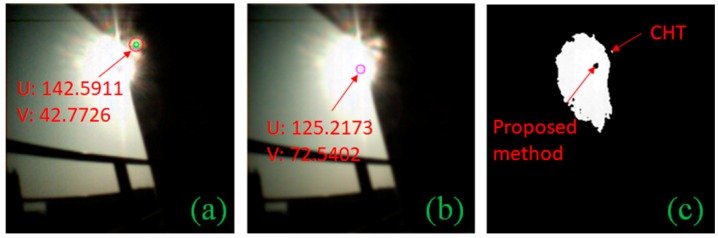
(**a**) Circle Hough transform (CHT) failing to detect the black sun; (**b**) the proposed method; (**c**) binary segmented image of the sun showing an irregular shape due to glare.

**Figure 7:**
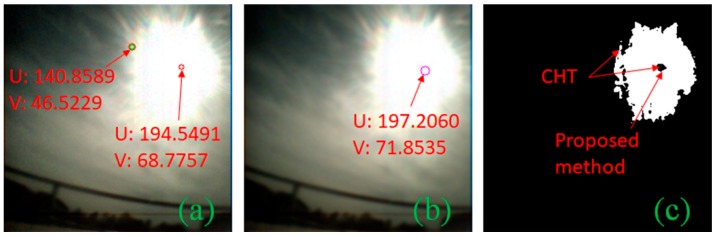
(**a**) CHT detecting multiple circles; (**b**) the proposed method; (**c**) binary segmented image of the sun capture with the black sun with glare.

In case of a MEMS-based sun sensor, their construction has a slit in front of APS which allows them to easily detect the sun centroid simply by finding out which pixels are illuminated. The problem of detecting centroid is prominent for camera-based sun sensors where other imaging artifacts are introduced. To avoid the glare when capturing the sun image, Minor et al. [20] and Rahim et al. [21] installed a neutral density filter in front of the lens to reduce the incoming light. Thus, allowing them to use CHT for centroid detection. Liu et al. [15] also used CHT to detect the sun centroid and was able to compensate for the blooming effect by using an aggressive threshold. We tested CHT against the proposed centroid detection algorithm (Figure 6b and Figure 7b). Two datasets were used for comparison; one with 467 images captured at 1 min intervals and a second with 551 images taken at 1 s intervals as shown in Table 1. Both dataset images were taken with the same camera at different durations and locations with varying illumination. CHT had a detection rate of only 9.85%, whereas our algorithm achieved 99.78% in the first dataset. In the second dataset, however, CHT managed to detect 16.33% of the time, but it detected other artifacts as circles too (Figure 7a). Our proposed method had a 99.82% detection rate in the dataset. The reason for failure of the CHT (Figure 6a) method can be summarized by looking at the binary segmented image in Figure 6c and Figure 7c; the segmented image shows that the sun image due to the glare appears irregular. Since we know that the black sun represents the true center of the sun, it implies that the center-of-mass of this irregular shape will not always appear to be the sun center. The black sun centroid-detection approach enables our sun vector extraction to cope with a light cloud cover and glare.

### 2.4. Sun Vector from Camera Pixels

**Figure 8:**
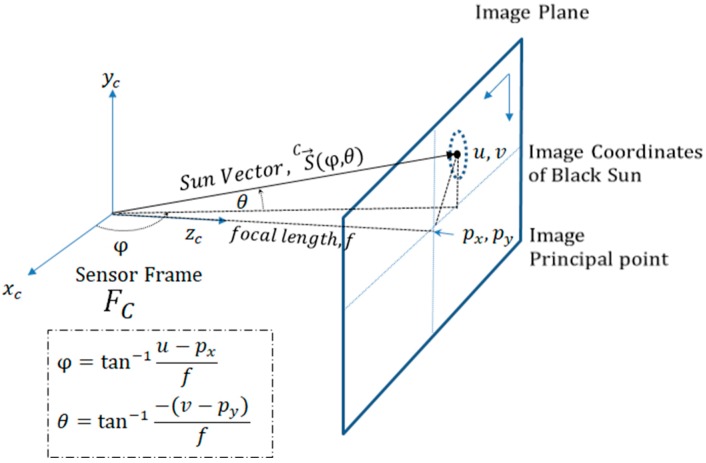
Conversion of pixel coordinates to sun vector representation.

For further calculations, we need to represent the centroid pixel coordinates as a sun vector $\vec{V}$ in terms of the azimuth angle φ and elevation angle *θ* in our sensor frame ($F_{C}$). We can estimate the sun vector using the calibrated camera’s intrinsic parameters [focal length *f*, principal point (*p_x_*, *p_y_*)] and the image coordinates (*u*, *v*) of the black sun centroid obtained in the image plane from image processing (Figure 8).

## 3. Stationary Application

### 3.1. Approach

**Figure 9:**
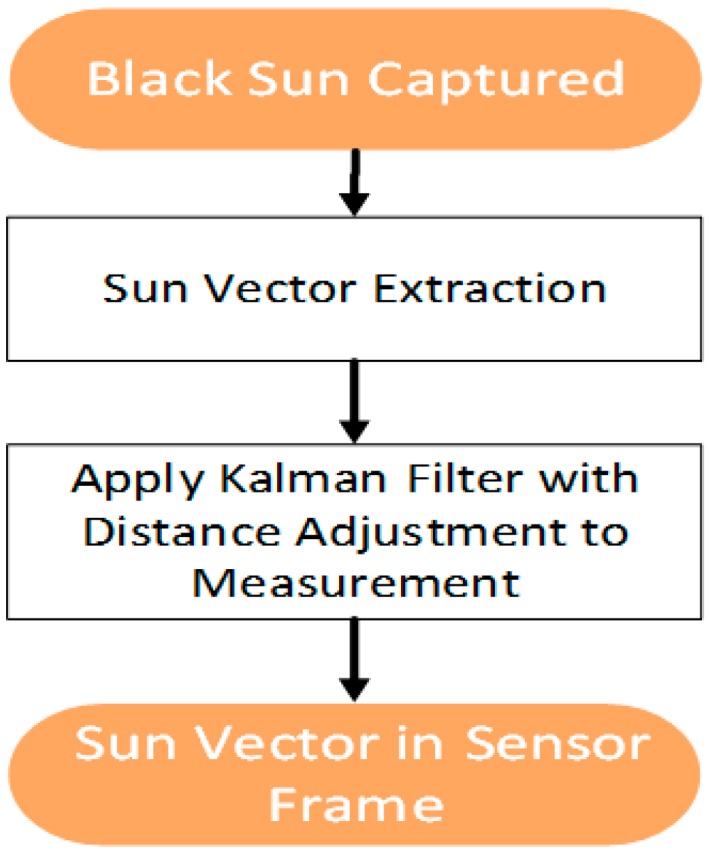
Stationary application process flow.

For a stationary application, we will employ a single image sensor configuration to capture the black sun. Owing to the stationary nature of the application, we can adopt a filtering scheme to the measurement coming from the centroid detection algorithm. This scheme would utilize the information of the slope from the ground truth of the pre-known location to remove the measurement noise. The process flow diagram is shown in Figure 9.

### 3.2. Filtering of the Sun Vector

The raw measurement generated from the proposed centroid-detection algorithm had inherent process noise due to the slight fluctuations in centroid estimation caused by the size and shape variations of the black sun. Therefore, to smooth out our measurement, we applied a noise filtering method such as the Kalman Filter (KF). The steps of KF are summarized below:

Prediction:

(1) $${\hat{x}}_{k}^{-}=Ax_{k-1}+Bu_{k-1}$$

(2) $$P_{k}^{-}=AP_{k-1}A^{T}+Q$$

Filtering:

(3) $$K_{k}=P_{k}^{-}H^{T}{\left(HP_{k}^{-}H^{T}+S\right)}^{-1}$$

(4) $${\hat{x}}_{k}={\tilde{x}}_{k}+K_{k}\left(z_{k}-H{\tilde{x}}_{k}\right)$$

(5) $$P_{k}=\left(I-K_{k}H\right)P_{k}^{-}$$

where k is the discrete time, A is the system matrix, B is the input matrix, $u_{k}$ is the input vector, $x_{k}$ is the state vector, ${\hat{x}}_{k}$ is the state estimate, $P_{k}^{-}$ is prediction error covariance,  H is the observation model, Q is the covariance matrix, $K_{k}$ is the Kalman gain, $z_{k}$ represents the sensor measurement and S is also error covariance. 

**Figure 10:**
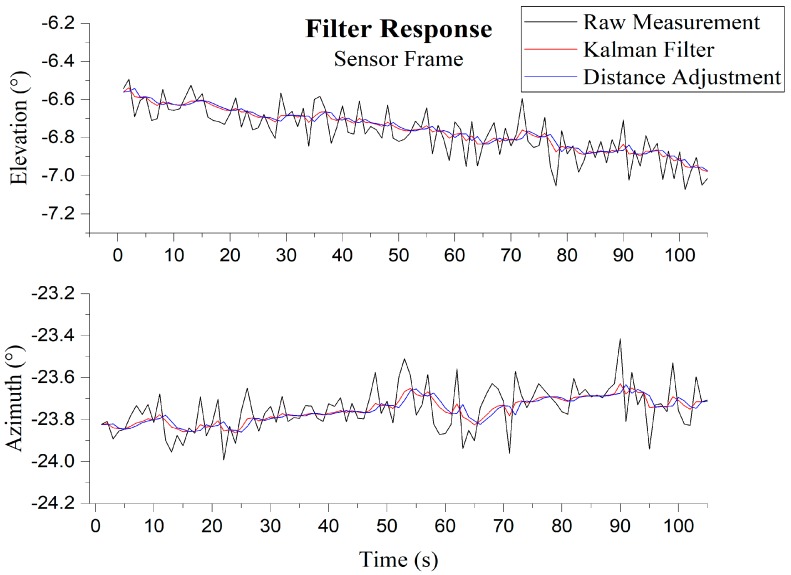
Effects of filtering of a single camera; **Top**: Elevation; **Bottom**: Azimuth.

We further improved the filtered vector by utilizing the distance between two consecutive sun vectors in the topocentric frame obtained from sun ephemeris data. For a pair of filtered measurement vectors ($x_{k}$ and $\;x_{k-1}$), the vector $x_{k-1}$ is adjusted such that the distance between vectors is equal to the distance obtained from two consecutive topocentric sun vectors. Figure 10 shows the effect of the filter in smoothing the raw measurement. The performance metrics are discussed in the experimentation section.

## 4. Non-Stationary Application

### 4.1. Approach

**Figure 11:**
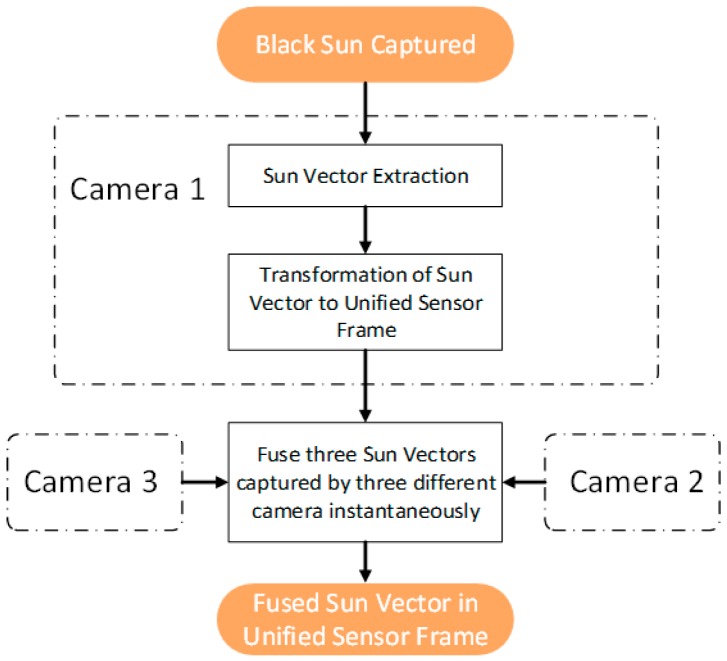
Non-Stationary application process flow.

For a non-stationary application, we cannot apply the filtering described previously, as the sensor would be moving. To address this, we employed a multiple-image-sensor icosahedron-based configuration. The icosahedron configuration allows three image sensors to capture the black sun simultaneously at any given time. Consequently, this sun sensor design will have a FOV of 360°. After the sun vector extraction, we used the sensor fusion method to fuse three vectors to provide an accurate and robust sun vector. The process flow diagram is shown in Figure 11.

### 4.2. Icosahedron Design

**Figure 12:**
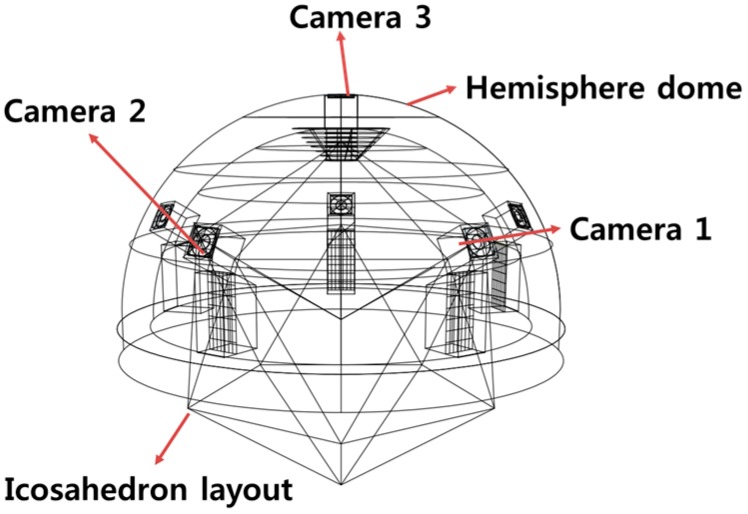
Multiple-image-sensor icosahedron configuration for the sun sensor design with a three-image sensor capable of capturing the sun simultaneously at any given time.

For measuring the sun vector with multiple image sensors sharing the same scene capture, we require hemispherical coverage. To meet this goal, we employed a dome structure based on an icosahedron configuration layout, or a similar geometric configuration approximating the spherical surface with planar surface patches of multiple cameras (six-camera configuration shown in Figure 12 as an example). An icosahedron configuration provides an optimal number of vertices facilitating equidistant camera placement. 

**Figure 13:**
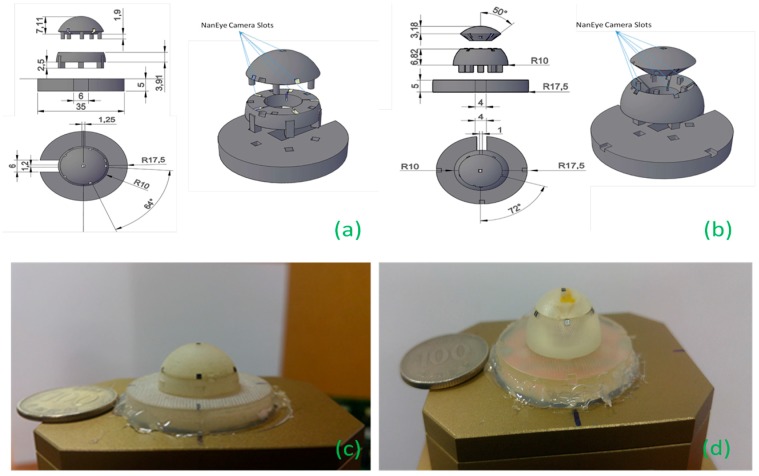
(**a**) and (**b**) CAD layouts of the first and second prototypes; (**c**) and (**d**) 3D printed sun sensor module with a coin for size comparison of the first and second prototypes.

**Figure 14:**
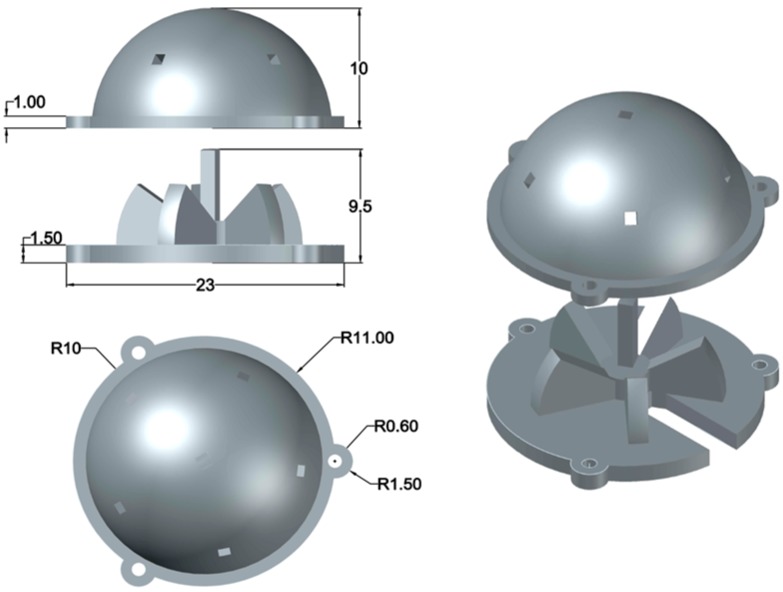
CAD layout of the aluminum metal design.

**Figure 15:**
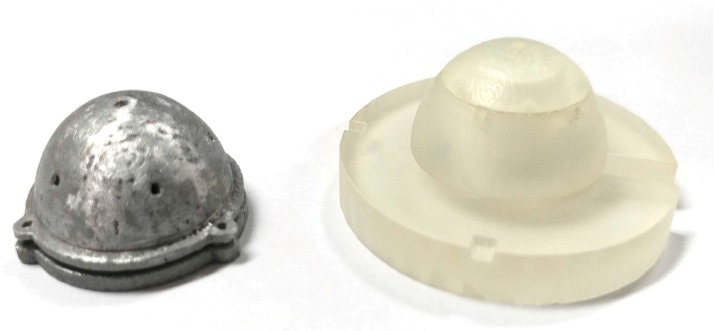
Aluminum metal design (**left**) alongside the second prototype (**right**).

Using the geometry of a polyhedron, specifically an icosahedron, we designed our sun sensor and positioned the cameras along the vertices of the polyhedron. Compensating for the offset from the center of the sphere by relating the parameters of the camera, we determined the solid angle made by the camera and then computed the ideal distance that would provide the optimal FOV intersection. The sensor CAD model was designed such that it could house six NanEye cameras and had an easy assembly. Initially, the angle between the cameras in the first prototype was 64°, as shown in Figure 13a,c, then later improved to 72° in the second prototype shown in Figure 13b,d after experimentation. To improve the solid angle between the cameras, one camera was perpendicular to the horizon whereas cameras were placed in a ring. The two prototypes were built in three parts to allow easy access and convenience in mounting. The footprint of the second prototype was 35 mm × 35 mm × 15 mm, with a weight of approximately 22.76 g with camera modules (without controller). Based on the second prototype, we adapted our design for aluminum metal printing and managed to reduce the size even further down to 23 mm × 23 mm × 11 mm. The computer-aided design (CAD) model can be seen in Figure 14, whereas the physical metal sun sensor case is shown along with the second prototype in Figure 15.

### 4.3. Individual Sensor Orientation Estimation

As we are measuring sun vector in multiple sensor frames, we need to transform them into a unified sensor frame for sensor fusion. Attitude determination problem is widely described by the Wahba’s problem [22] involving a multiple numbers of vectors observations. The problem (Equation (6)) is to find the orthogonal matrix A between two sets of corresponded unit vector $b_{i}$ and $r_{i}$ by minimizing the loss function;

(6) $$L\left(A\right)=\frac{1}{2}\overset{N}{\underset{i=1}{\sum }}\|b_{i}-Ar_{i}\|{}^{2}$$

We will be using a computationally efficient algorithm developed by Lourakis [23], which establishes links between attitude estimate and absolute orientation. The absolute orientation problem tries to find the Euclidean transformation  R, t  that aligns two sets of corresponding 3D points $p_{i}$ and $q_{i}$ measured in two different coordinate systems by a least squares solution minimizing the mean squared residual error (Equation (7)). 

(7) $$\frac{1}{2}\overset{N}{\underset{i=1}{\sum }}\|q_{i}-{\left(Rp_{i}+t\right)\|}^{2}$$

### 4.4. Sensor Fusion

As mentioned, the sun sensor with an icosahedron configuration allowed us to capture three sun vectors simultaneously. For non-stationary applications, we are going to fuse the vectors to obtain an accurate and robust sun vector. We used the Covariance Projection Method (CPM) [24,25] for fusing our sensors data in a unified sensor frame. The CPM is based on projecting the joint probability distribution of redundant data sources onto the constraint manifold. The constraint manifold represents the constraints to be satisfied among the redundant data sources, which is defined in the extended space with all the redundant sources of data considered as independent variables. Then, the CPM framework of data fusion represents the projected probability distribution on the constraint manifold as the result of data fusion. The individual camera covariance can be assigned by the variances obtained from experimentation when running the cameras in a stationary application.

## 5. Experimentation

We conducted our experiments under the open sky at the location 37° 17’ 34.17” latitude and 126° 58’ 41.754” longitude, but without heavy cloud cover to avoid any illumination issue due to clouds. The ground truth solar angles were obtained from equations provided by NOAA/ESRL’s Global Monitoring Division (GMD) [26] based on Jean M.’s book on astronomical algorithms [27] with a high degree of accuracy. The equations inputs are the GPS coordinates of the sun sensor and the time of the experiment. Even though the cameras were capable of providing 42 to 55 fps, but in the absence of a hardware trigger, we throttled the image grab at 1-s intervals for the experimentation. Nearly 35,000 images were taken that captured the black sun effect. The proposed centroid-detection algorithm takes an average of 85 ms where the maximum is 120 ms, and the minimum is 60 ms. 

### 5.1. Black Sun Effect on the Error of Sun Vector Measurement

**Figure 16:**
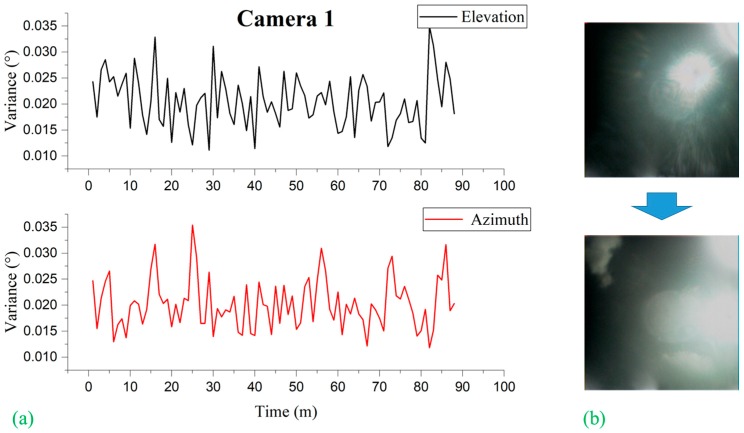
(**a**) Camera 1 variances observed by elevation and azimuth during experimentation subdivided at 1-minute intervals; (**b**) image frames showing the transition of the position of sun image captured on image plane from beginning to end of experimentation.

**Figure 17:**
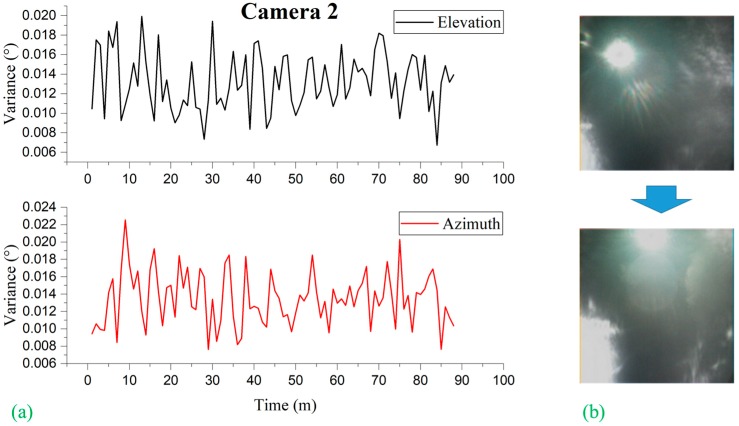
(**a**) Camera 2 variances observed by elevation and azimuth during experimentation subdivided at 1-minute intervals; (**b**) image frames showing the transition of the position of sun image captured on image plane from beginning to the end of experimentation.

**Figure 18:**
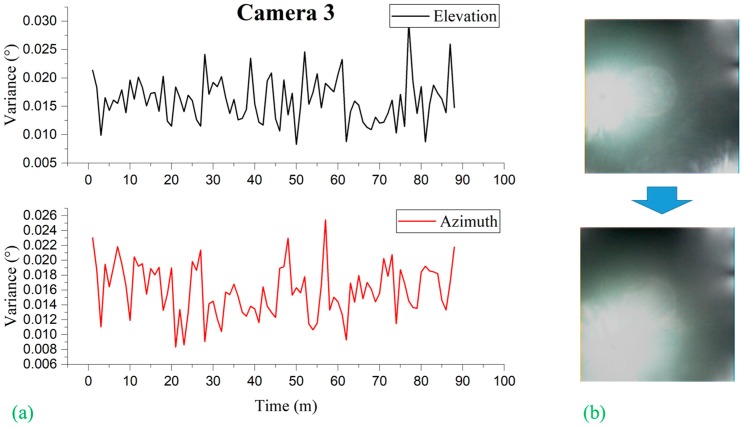
(**a**) Camera 3 variances observed by elevation and azimuth during experimentation subdivided at 1 min intervals; (**b**) image frames showing the transition of the position of sun image captured on image plane from beginning to the end of experimentation.

Liu et al. [15] observed that, for conventional image-based sun sensors, the error or the variance in the sun vector measurement is subject to the location of the sun captured on the image plane. They limited the measurement only around the image center due to the growing effect of distortion on the measurement accuracy when moving to the edge of the image. It is interesting to note that, in our experiment, we observed no such phenomena: No distortion effect is observed in relation to the location of the sun image due to the use of the black sun. In other words, the error or the variance in the sun vector measurement is independent of the measurement location on the image plane, as shown in Figure 16, Figure 17, and Figure 18. Figure 16, Figure 17, and Figure 18 represent the variation of variances measured during 90 minutes of the experiment for cameras 1, 2 and 3, respectively, of different orientations. The variances were computed at every 1-minute interval based on the sun vector measurement from each second. Without having the ground truth of the camera coordinate frame with reference to the topocentric frame, the variance statistics were based on the angle difference between two consecutive sun vector measurements the ground truth of which is known.

### 5.2. Stationary Application

**Figure 19:**
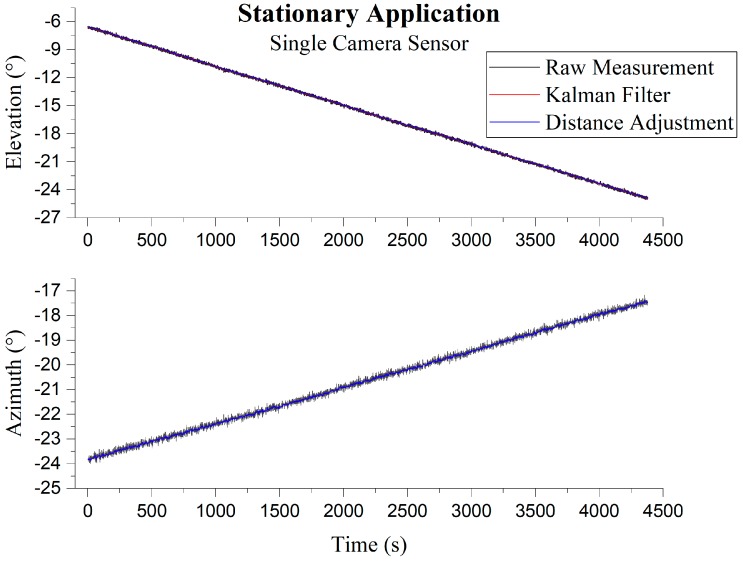
Performance observed in the stationary application by a single camera configuration; **Top**: Elevation; **Bottom**: Azimuth.

The following root-mean-square-errors (RMSEs) along with standard error given in Table 2 are in degrees (°) and were calculated from the constant ground truth sun vector 1−s angle difference, and the measured angle difference 1-s between two consecutive measurements. Without filtering the azimuth angle, the best RMSE and standard error for the given sample, in either case, was 0.1250° (0.0884°), whereas the elevation angle RMSE was 0.1255° (0.0888°). Figure 19 visually highlights the importance of filtering after measurement, as the error in raw measurement is asymmetrical. After filtering, it can be seen that this reduces the azimuth angle RMSE and standard error to 0.0179° (0.0127°) and elevation angle RMSE and standard error to 0.0184° (0.0130°). KF alone exhibits around 83.59% improvement in error whereas combined with distance adjustment, the improvement was 85.68%.

### 5.3. Non-Stationary Application

**Figure 20:**
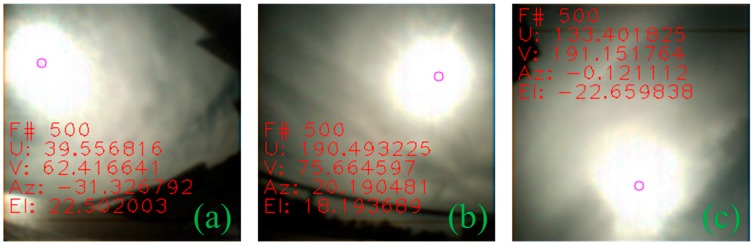
Images captured simultaneously by three cameras in the icosahedron configuration; (**a**) camera 1; (**b**) camera 2; (**c**) camera 3.

**Figure 21:**
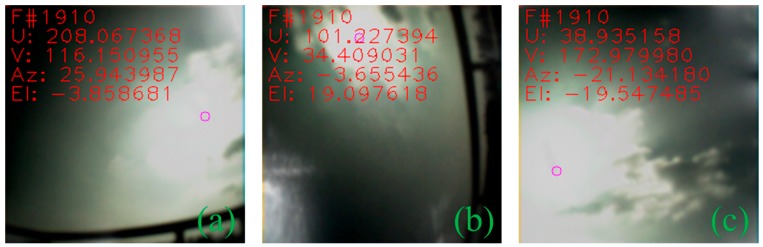
Images captured simultaneously under dynamic cloud condition by three cameras in the icosahedron configuration; (**a**) camera 1; (**b**) camera 2; (**c**) camera 3.

**Figure 22:**
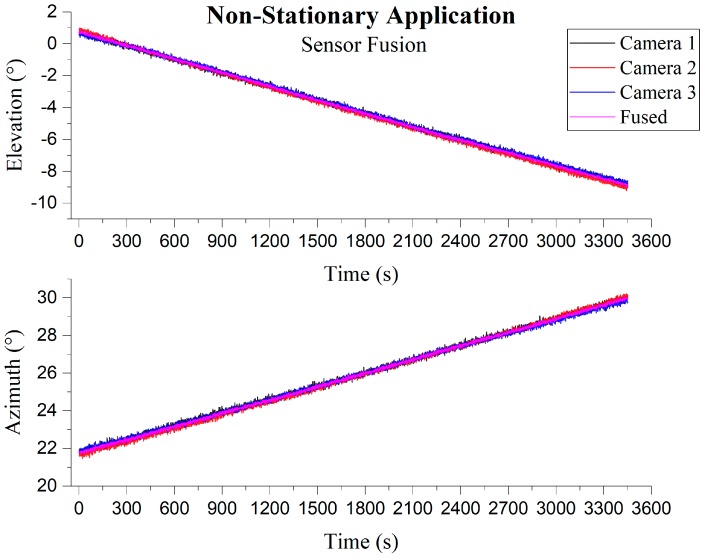
Three-camera sun vector reading with the fused vector in a non-stationary application; **Top**: Elevation; **Bottom**: Azimuth.

**Figure 23:**
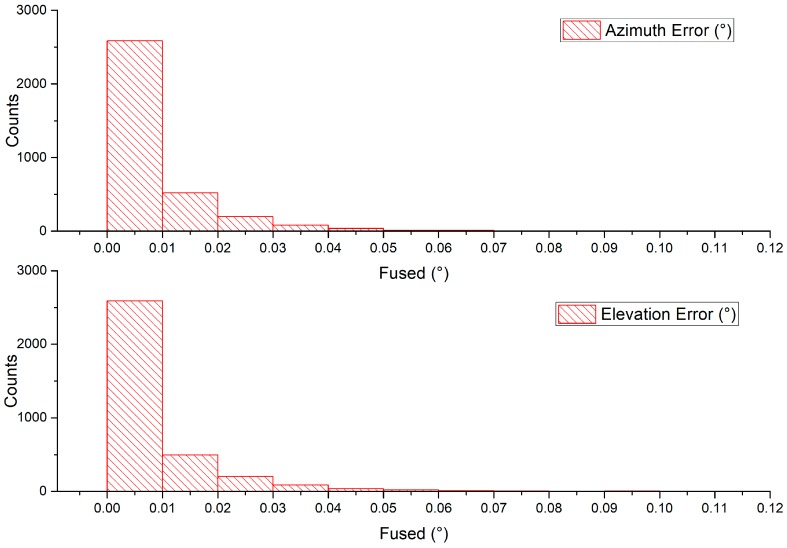
Error distribution seen by the multiple-camera configuration in the non-stationary application.

In the case of non-stationary measurement, at least three cameras will capture the sun simultaneously. Firstly, each camera measurement was transformed into a unified sensor frame (camera 1 frame in this case), and then the measurements were fused together using sensor fusion to obtain the result. Camera covariances were assigned based on experimentation result from running the individual camera in a stationary application. Figure 20 and Figure 21 shows images captured by individual cameras with sensor frame information overlay, and the RMSEs along with standard error calculated from time synchronized samples which are given in Table 3. The graphs in Figure 22 compare individual cameras and fusion results in a unified sensor frame. By fusing, not only did it come close to the ground truth, but it also stabilized the overall measurements. The fused sun measurement shows an RMSE and standard error in the azimuth and elevation angles as 0.0713° (0.0504°) and 0.0717° (0.0507°), respectively. The error distribution is shown in Figure 23. 

## 6. Conclusions

In this paper, we explained in depth how we took advantage of the phenomenon in CMOS image sensors known as the “black sun” caused by electron overspill at an oversaturated pixel. It allows the extraction of the sun centroid accurately and robustly even when the sun image appears irregular and noisy due to glare. Compared to other image-based sun sensors, we observed that black sun based sun vector measurement error or variance is independent of the location of the sun captured on the image plane. We demonstrated the performance of our approach in two applications using micro-camera as small as 1 mm × 1 mm × 1.74 mm in size and with a pixel resolution as low as 250 × 250. First, a stationary application (e.g., solar panel or heliostat) where we observed an accuracy of 0.0127° and 0.0130° in azimuth and elevation angles, respectively, with an FOV of 90° and second, a non-stationary application for a moving platform with six-image-sensors in icosahedron geometry of 23 mm × 23 mm × 12 mm in size, which showed an accuracy of 0.0504° and 0.0507° in the azimuth and elevation angles, respectively, with an FOV of 360°. The fusion of multiple image-sensors allows us to improve the results as compared to an individual sensor while at the same time use a minimal number of image sensors to achieve hemispherical coverage. In further studies, we aim to improve the speed and accuracy of the sensor.

## Figures and Tables

**Table 1:** Detection Rate.

Positive Detection (%)	Circular Hough Transform	Our Method
Dataset 1	9.8501	99.7858
Dataset 2	16.3339	99.8185

(Tested on Dataset 1 with 467 images and dataset 2 with 551 images).

**Table 2:** Stationary Application Single Camera Sensor RMSE.

RMSE (Standard. Error)	Azimuth	Elevation
Raw Measurement	0.1250° (0.0884°)	0.1255° (0.0888°)
Kalman Filter	0.0205° (0.0145°)	0.0208° (0.0147°)
Distance Adjustment	0.0179° (0.0127°)	0.0184° (0.0130°)

**Table 3:** Non-Stationary Application root-mean-square (RMS) Error.

RMS Error (Standard Error)	Azimuth	Elevation
Camera 1	0.1429° (0.1010°)	0.1422° (0.1005°)
Camera 2	0.1158° (0.0819°)	0.1095° (0.0775°)
Camera 3	0.1261° (0.0892°)	0.1268° (0.0897°)
Fused	0.0713° (0.0504°)	0.0717° (0.0507°)
